# Paroxysmal motor signs in multiple sclerosis: an illustrative videotaped case

**DOI:** 10.1093/omcr/omag016

**Published:** 2026-03-23

**Authors:** Ghita Lahmam, Asmae Sikkal, Hajar Khattab, Kamal Haddouali, Salma Bellakhdar, Bouchra El Moutawakil, Mohamed Abdoh Rafai, Hicham El Otmani

**Affiliations:** Hassan II University of Casablanca, Faculty of Medicine and Pharmacy of Casablanca, Rue Tarik Ibn Ziad, Casablanca, Casablanca-Settat 20100, Morocco; Department of Neurology, CHU Ibn Rochd, Boulevard des Hôpitaux, Quartier des Hôpitaux, Casablanca-Settat 20250, Casablanca, Morocco; Hassan II University of Casablanca, Faculty of Medicine and Pharmacy of Casablanca, Rue Tarik Ibn Ziad, Casablanca, Casablanca-Settat 20100, Morocco; Department of Neurology, CHU Ibn Rochd, Boulevard des Hôpitaux, Quartier des Hôpitaux, Casablanca-Settat 20250, Casablanca, Morocco; Hassan II University of Casablanca, Faculty of Medicine and Pharmacy of Casablanca, Rue Tarik Ibn Ziad, Casablanca, Casablanca-Settat 20100, Morocco; Department of Neurology, CHU Ibn Rochd, Boulevard des Hôpitaux, Quartier des Hôpitaux, Casablanca-Settat 20250, Casablanca, Morocco; Hassan II University of Casablanca, Faculty of Medicine and Pharmacy of Casablanca, Rue Tarik Ibn Ziad, Casablanca, Casablanca-Settat 20100, Morocco; Department of Neurology, CHU Ibn Rochd, Boulevard des Hôpitaux, Quartier des Hôpitaux, Casablanca-Settat 20250, Casablanca, Morocco; Hassan II University of Casablanca, Faculty of Medicine and Pharmacy of Casablanca, Rue Tarik Ibn Ziad, Casablanca, Casablanca-Settat 20100, Morocco; Department of Neurology, CHU Ibn Rochd, Boulevard des Hôpitaux, Quartier des Hôpitaux, Casablanca-Settat 20250, Casablanca, Morocco; Laboratory of Genetics and Molecular Pathology, Faculty of Medicine and Pharmacy, Hassan II University of Casablanca, Rue Tarik Ibn Ziad, Casablanca-Settat 20100, Casablanca, Morocco; Hassan II University of Casablanca, Faculty of Medicine and Pharmacy of Casablanca, Rue Tarik Ibn Ziad, Casablanca, Casablanca-Settat 20100, Morocco; Department of Neurology, CHU Ibn Rochd, Boulevard des Hôpitaux, Quartier des Hôpitaux, Casablanca-Settat 20250, Casablanca, Morocco; Laboratory of Genetics and Molecular Pathology, Faculty of Medicine and Pharmacy, Hassan II University of Casablanca, Rue Tarik Ibn Ziad, Casablanca-Settat 20100, Casablanca, Morocco; Hassan II University of Casablanca, Faculty of Medicine and Pharmacy of Casablanca, Rue Tarik Ibn Ziad, Casablanca, Casablanca-Settat 20100, Morocco; Department of Neurology, CHU Ibn Rochd, Boulevard des Hôpitaux, Quartier des Hôpitaux, Casablanca-Settat 20250, Casablanca, Morocco; Research Laboratory on Nervous System Diseases, Neurosensory Diseases and Disability, Faculty of Medicine and Pharmacy, Hassan II University of Casablanca, Rue Tarik Ibn Ziad, Casablanca-Settat 20100, Casablanca, Morocco; Hassan II University of Casablanca, Faculty of Medicine and Pharmacy of Casablanca, Rue Tarik Ibn Ziad, Casablanca, Casablanca-Settat 20100, Morocco; Department of Neurology, CHU Ibn Rochd, Boulevard des Hôpitaux, Quartier des Hôpitaux, Casablanca-Settat 20250, Casablanca, Morocco; Laboratory of Genetics and Molecular Pathology, Faculty of Medicine and Pharmacy, Hassan II University of Casablanca, Rue Tarik Ibn Ziad, Casablanca-Settat 20100, Casablanca, Morocco

**Keywords:** ataxia, dysarthria, Choreo-dystonia, paroxysmal, multiple sclerosis

## Abstract

Multiple sclerosis (MS) presents with a wide spectrum of neurological manifestations, among which paroxysmal motor phenomena remain a diagnostic and therapeutic challenge. We report the case of a 29-year-old male diagnosed with relapsing–remitting MS (RRMS) who exhibited episodes of paroxysmal dystonia, dysarthria-ataxia, and ptosis. Brain MRI revealed multiple demyelinating plaques, including a contrast-enhancing lesion in the right midbrain, which provides an anatomical correlation with the patient's neurological manifestation. The dystonic movements resolved with corticosteroid therapy, while symptomatic management with carbamazepine was required for dysarthria-ataxia. Ocrelizumab has been proposed as a long-term disease-modifying treatment. This case highlights the importance of recognizing paroxysmal motor signs in MS for early diagnosis and tailored management, despite their elusive pathogenesis.

## Introduction

Multiple sclerosis (MS) is a chronic autoimmune disorder characterized by a diverse and often unpredictable range of neurological manifestations. Among these, paroxysmal motor signs are uncommon yet intriguing phenomena that pose both diagnostic and therapeutic challenges. These episodic motor disturbances, including paroxysmal dystonia, dysarthria-ataxia, and tonic spasms, may significantly impact patients' quality of life and often lead to diagnostic confusion, particularly when occurring as the initial presentation. Despite extensive research, the underlying pathophysiological mechanisms remain incompletely understood.

We present a case of a patient diagnosed with RRMS, exhibiting recurrent episodes of paroxysmal dystonia, dysarthria-ataxia, and ptosis, documented through video imaging. This case illustrates the importance of recognizing these transient motor phenomena and explores their likely anatomical correlates within demyelinating lesions.

## Case report

**Figure 1:**
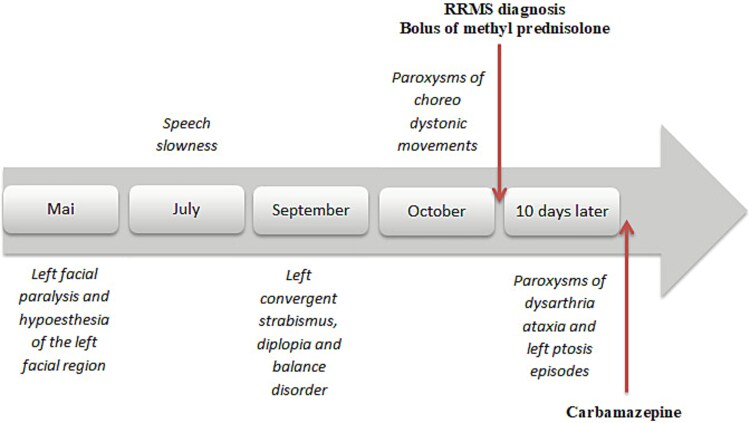
This timeline summarizes clinical relapses of the patient.

A 29-year-old male with no prior medical history presented with a relapsing–remitting neurological syndrome (Fig. 1). The initial episode, occurring in May 2024, consisted of left-sided facial paralysis and hypoesthesia, which resolved spontaneously within 20 days. The second relapse, in July 2024, manifested as speech slowness, spontaneously resolving within one week. The third episode, in September 2024, involved left convergent strabismus, diplopia, and balance impairment, also resolving within 20 days.

**Figure 2:**
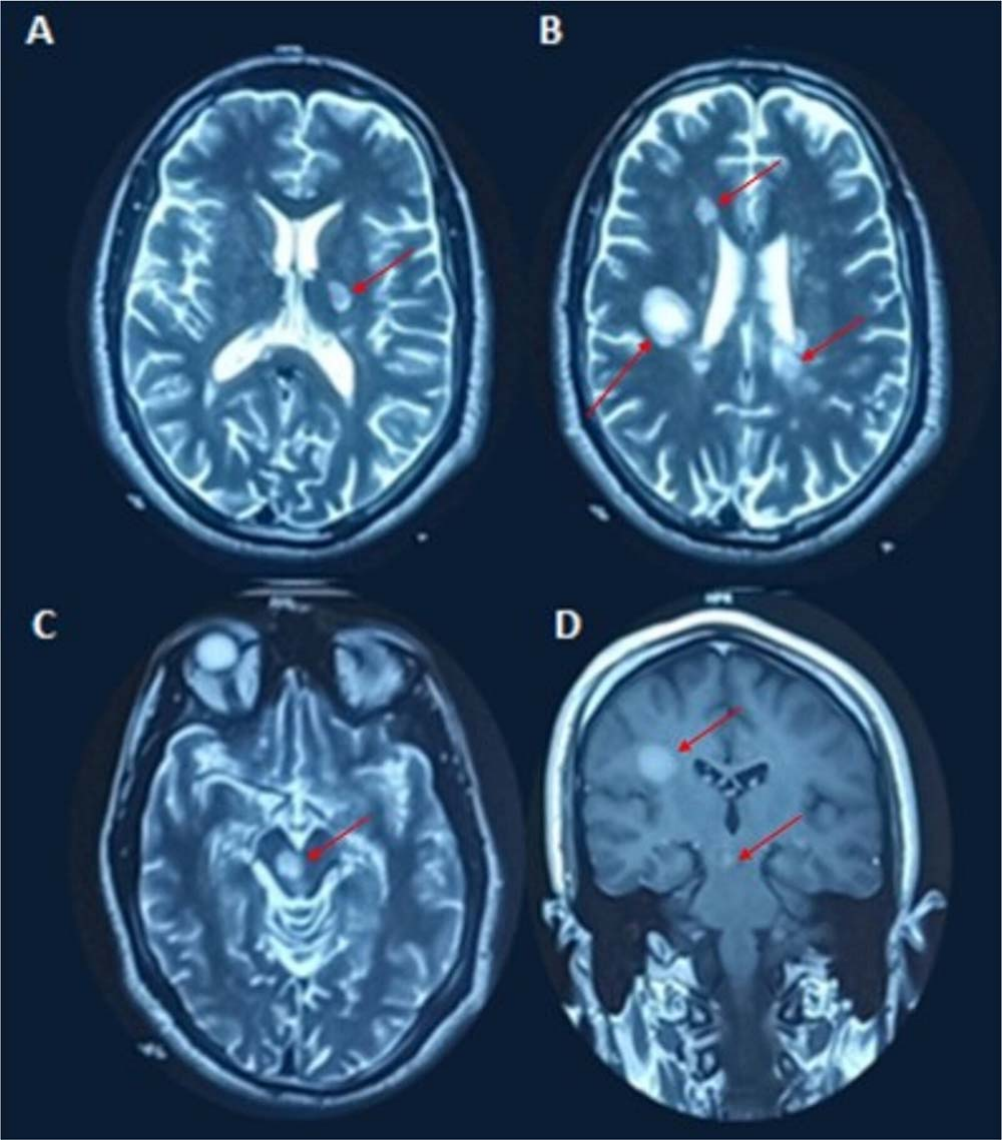
(A) Axial T2-weighted MRI showing a hyperintense lesion involving the posterior limb of the left internal capsule. (B) Axial T2-weighted MRI demonstrating multiple periventricular and juxtacortical hyperintense lesions, including a right semioval center lesion with a pseudo-tumoral appearance. (C) Axial T2-weighted MRI showing a hyperintense lesion in the right midbrain. (D) Coronal contrast-enhanced MRI revealing gadolinium enhancement of the right semioval center lesion and the right midbrain, consistent with active inflammatory demyelination.

The most recent and striking episode, in October 2024, involved dozens of daily paroxysms of hyperkinetic choreo-dystonic movements affecting the left hemibody, including the face. These brief, non-painful, and consciousness-preserved paroxysms occurred spontaneously or were triggered by voluntary movements (Video 1). Brain MRI (Fig. 2) revealed multiple demyelinating plaques, notably in the right midbrain, periventricular white matter, right semioval center (with pseudo-tumoral appearance), and the posterior limb of the left internal capsule, with contrast enhancement observed in the right midbrain and semioval center. Cerebrospinal fluid (CSF) analysis showed intrathecal IgG synthesis (IgG index 0.79) and oligoclonal bands. Based on McDonald criteria, a diagnosis of relapsing–remitting MS (RRMS) was established. Other autoimmune disorders, including neuro-Behçet disease, were also carefully excluded based on the absence of oral or genital aphthosis, pseudofolliculitis, ocular involvement on ophthalmological examination, and a negative autoimmune and inflammatory workup. A five-day course of intravenous methylprednisolone led to the resolution of dystonic paroxysms; however, 10 days later, the patient developed recurrent episodes of paroxysmal dysarthria-ataxia and left ptosis, lasting less than a minute and triggered by hyperventilation (Video 2). Symptomatic treatment with carbamazepine (600 mg/day) was initiated and maintained for three months, leading to marked clinical improvement and near-complete disappearance of paroxysms. Ocrelizumab was proposed as a disease-modifying treatment to prevent further relapses.

## Discussion

The paroxysmal symptoms observed in multiple sclerosis include dystonia, ataxia, tonic spasms, paresthesia, akinesia, and pain-related syndromes such as trigeminal neuralgia. Overall, movement disorders are relatively uncommon, reported in approximately 1%–4% of patients [1]. Tremor is the most frequent movement disorder, affecting up to 25–58% of patients depending on disease duration and severity. In contrast, paroxysmal motor phenomena, including paroxysmal dystonia and tonic spasms, are much rarer, with an estimated prevalence of less than 1%–2% [2]. Paroxysmal dysarthria–ataxia is exceptional and has been reported only sporadically in case reports and small series. These transient motor disturbances, although uncommon, can severely impact daily function and are frequently misdiagnosed as epileptic phenomena or functional disorders.

A summary of some previously reported cases is presented in Table 1, detailing patient age, type of movement disorder, initial symptoms, lesion localization, triggers, treatment, and outcome.

**Table 1:** Paroxysmal motor phenomena as inaugural or early manifestations of multiple sclerosis (case reports and small series).

Reference	Type of Movement Disorder	Initial Presentation	MRI Lesion Location	Treatment	Outcome
Berger et al. 2004^a^	Paroxysmal dystonia	Initial paroxysmal dystonia	Various	Antiepileptics	Diagnosis sometimes delayed, later MS evolution
Florczak & Kozubski 2003^b^	Paroxysmal dystonia	Initial paroxysmal dystonia	Midbrain and other plaques	Carbamazepine + corticosteroids	Response to carbamazepine, no relapse
Li 2010^c^	Paroxysmal dysarthria–ataxia	Paroxysmal dysarthria or dysarthria–ataxia	Around red nucleus	Carbamazepine	Good response
Iorio et al. 2014^d^	Paroxysmal ataxia & dysarthria	Paroxysmal dysarthria + ataxia	Midbrain lesion	Carbamazepine	Complete resolution
Al Dehailan 2019^e^	Paroxysmal dystonia/tonic spasms	Initial paroxysmal dystonia	Not specified	Carbamazepine + DMT	Durable improvement
Planas Ballvé 2022^f^	Paroxysmal kinesigenic dyskinesia	Paroxysmal movements as sole symptom	Not specified	Carbamazepine	Complete resolution
Díaz del Arco 2024^g^	Paroxysmal dystonia/tonic spasms	Tonic paroxysmal spasms as initial symptom	Not specified	IV corticosteroids + CBZ	Rapid disappearance of spasms
El Otmani et al. 2014^h^	Paroxysmal dystonia	Bilateral episodic dystonia	Internal capsule	Corticosteroids	Complete resolution
E. Ciampi et al. 2017^i^	Secondary paroxysmal dyskinesia	Short paroxysmal dyskinesias	Thalamus, midbrain, cerebellar peduncles	Acetazolamide/Levetiracetam	Improvement

^a^Berger JR, Sheremata WA, Melamed E. Paroxysmal dystonia as the initial manifestation of multiple sclerosis*.* JAMA Neurol. 2004.

^b^Florczak J, Kozubski W. Paroxysmal focal dystonia as the initial manifestation of multiple sclerosis: case report*.* Neurol Neurochir Pol. 2003;37 Suppl 5:127–131.

^c^Li S. Paroxysmal dysarthria and ataxia in multiple sclerosis and corresponding MRI findings*.* J Neurol. DOI:10.1007/s00415-010-5748-4.

^d^Iorio R, Capone F, Plantone D, Batocchi AP. Paroxysmal ataxia and dysarthria in multiple sclerosis. J Clin Neurosci. 2014;21 (1):174–175. doi:10.1016/j.jocn.2013.01.031.

^e^Al Dehailan AS. Paroxysmal dystonia as the initial presentation of multiple sclerosis posing a diagnostic challenge*.* Neurosciences (Riyadh). 2019;24 (3):236–239.

^f^Planas Ballvé A, Caballol N, Gubieras L, Cardona X, Gómez Ruiz I, Balagué Marmaña M, Ávila A. Paroxysmal Kinesigenic Dyskinesia Secondary to Multiple Sclerosis. Am J Biomed Sci & Res. 2022;16 (1):Article 002187. doi:10.34297/AJBSR.2022.16.002187.

^g^Díaz del Arco C. Paroxysmal dystonia presenting as the first sign of multiple sclerosis: case report*.* Mult Scler Relat Disord. 2024;92:106053.

^h^El Otmani H, Benmansour Y, Araqi-Houssaini A, Benkirane N, Dany F, Rafai MA, et al. Dystonie paroxystique et sclérose en plaques. Revue Neurologique. 2014; 170: *119–123.*

^i^Ciampi E, Uribe-San-Martín R, Godoy-Santín J, Cruz JP, Cárcamo-Rodríguez C, Juri C. Secondary paroxysmal dyskinesia in multiple sclerosis: Clinical-radiological features and treatment. Case report of seven patients. Mult Scler. 2017 Nov;23(13):1791–1795. 10.1177/1352458517702968. Epub 2017 Apr 11. PMID: 28397579.

Paroxysmal dystonia is typically stereotyped, lasting from 30 s to 5 min, and may occur up to 100 times per day over weeks [3]. Various triggers, including hyperventilation, voluntary movement, tactile stimulation, or emotional stress, have been identified [4].

**Figure 3:**
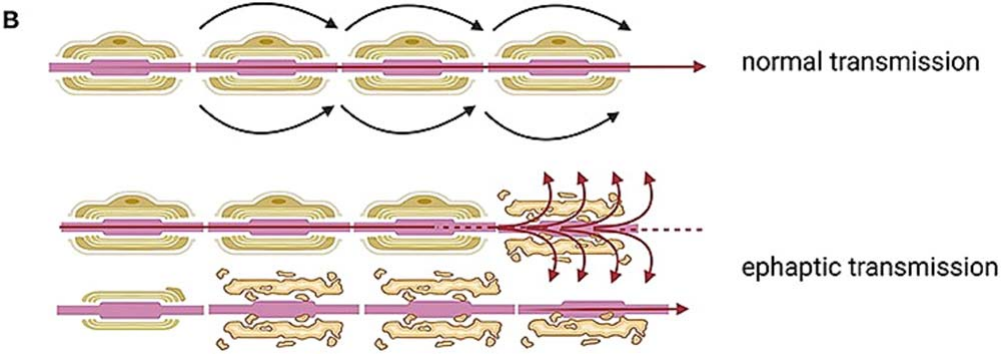
Schematic representation of ephaptic transmission in demyelinated axons, adapted from Bruno et al., theoretical and therapeutic implications of the spasticity-plus syndrome model in multiple sclerosis, Frontiers in neurology 2022, under creative commons attribution (CC BY) license.

The proposed mechanism of paroxysmal motor phenomena in multiple sclerosis involves nonsynaptic ephaptic transmission [2], which occurs when a demyelinated axon abnormally conducts an action potential that spreads to an adjacent fiber without involving a synapse. In healthy axons, the myelin sheath electrically isolates nerve fibers, preventing such cross-activation. However, demyelination allows current to ‘leak’ into neighboring axons, depolarizing them and triggering involuntary, brief, and stereotyped motor discharges. This mechanism explains the sudden onset, short duration, and high frequency of paroxysmal dystonia, tonic spasms, and dysarthria–ataxia in MS. Demyelinating lesions located along the corticospinal tract, brainstem, basal ganglia, or semioval center can thus produce these transient motor symptoms through ephaptic activation [5]. (Fig. 3) [6].

In our patient’s case, the choreo-dystonic movements were likely due to the right mesencephalic lesion, which showed contrast enhancement on MRI, indicating an active inflammatory process. Given that the subthalamic nucleus is anatomically located just above this mesencephalic lesion, it is plausible that its involvement contributed to the hyperkinetic nature of the movement disorder. This differs from the most common paroxysmal movement disorder in MS, which is tonic spasm. The latter is topographically linked to corticospinal tract involvement, regardless of the level of the lesion, though it is particularly frequent when affecting the posterior limb of the internal capsule or the spinal cord, which is closely resembles the tonic spasms observed in the neuromyelitis optica spectrum disorders (NMOSD).

Paroxysmal dystonia in MS may respond to corticosteroid therapy, as seen in our patient. However, treatment responses vary, and additional therapies such as acetazolamide, carbamazepine, other anticonvulsant drugs [1] may be necessary. In refractory cases, botulinum toxin injections have been reported as beneficial [7]. Spontaneous disappearance of symptoms is possible.

Paroxysmal dysarthria-ataxia (PAD) is a transient phenomenon, first described by Parker in 1946 [8]. It has been reported rarely in strokes and Behçet disease. While ataxia is a recognized feature of MS, isolated PAD remains rare and is more frequently associated with brainstem involvement.

PAD episodes can be triggered by hyperventilation or stress and are believed to result from ephaptic transmission in demyelinated axons, typically affecting the cerebello-thalamo-cortical pathways [9] in the medial midbrain in or above red nuleus.

In our patient’s case, the right midbrain lesion strongly correlates with PAD, and its delayed onset after corticosteroid treatment may be linked to the resolution of perilesional edema. Similarly, the contralateral ptosis may be explained by bilateral innervation of the levator palpebrae superioris subnucleus.

Carbamazepine, Levetiracetam, Lamotrigine, Lacosamide and Phenytoin [10] have been shown to be effective in treating PAD. Antiepileptic drugs are usually administered for weeks to several months and can be tapered after sustained remission, as paroxysmal symptoms in MS are often transient [1].

## Conclusion

Paroxysmal signs in MS are uncommon yet clinically significant and should be recognized by neurologists to facilitate early diagnosis and appropriate management. Although their pathophysiology remains incompletely understood, the anatomical correlations observed in our case support a role for demyelinating lesions in the midbrain in both choreo-dystonia and PAD. Symptomatic treatment with antiepileptic drugs remains the cornerstone of therapy, while corticosteroids may accelerate symptom resolution in inflammatory lesions. Further studies are needed to better elucidate the mechanisms underlying these fascinating paroxysmal manifestations in MS.

## Supplementary Material

video_1_omag016

video_2_omag016
